# Secular trend of mortality and incidence of rheumatoid arthritis in global ,1990–2019: an age period cohort analysis and joinpoint analysis

**DOI:** 10.1186/s12890-023-02594-2

**Published:** 2023-09-22

**Authors:** Fangyuan Yu, Hongwei Chen, Qi Li, Mengjun Tao, Ziyi Jin, Linyu Geng, Lingyun Sun

**Affiliations:** 1 School of Medicine, Southeast University, Nanjing, China; 2 Department of Rheumatology and Immunology, Nanjing Drum Tower Hospital, the Affiliated Drum Tower Hospital of Nanjing University Medical School, Nanjing, 210008 China; 3 Department of Rheumatology and Immunology, Nanjing Drum Tower Hospital Clinical College of Nanjing University of Chinese Medicine, Nanjing, China; 4 Department of Health Management Center, Yijishan Hospital, The First Affiliated Hospital of Wannan Medical College, Wuhu, China

**Keywords:** Rheumatoid arthritis, Disease burden, Disability adjusted life year, Age-period-cohort analysis

## Abstract

**Background:**

Rheumatoid arthritis (RA) is a major public health problem. Unfortunately, there is a scarcity of comprehensive and up-to-date information regarding the burden of RA and its dynamic trends in subsequent years. To examine the changing trends in the global burden of RA and forecast for 2044, which will facilitate the development of strategies tailored to RA burden and provide reference for the development of effective treatment guidelines.

**Methods:**

Following the general analytical strategy used the Global Burden of Disease Study (GBD) 2019, which included 204 countries, the age-standardized incidence rate (ASIR), age-standardized mortality rate (ASMR) and age-standardized disability adjusted of life year (DALY) rate for RA were analyzed.

**Results:**

The ASIR, ASMR and age-standardized DALY rate for RA in 2019 were 13.001/100,000 (95% UI, 11.833 ~ 14.274), 0.574/100,000 (95% UI, 0.356 ~ 0.793) and 39.565/100,000 (95% UI, 49.529 ~ 30.508), respectively. America had the highest ASIR [18.578(95% UI, 17.147 ~ 20.148)] and age-standardized DALY rate [53.676(95% UI, 40.106 ~ 67.968)] in 2019. Asia had the highest ASMR [0.681(95% UI, 0.802 ~ 0.480)] in 2019. From 1990 to 2019, a significant average annual percentage change (AAPC) in the ASIR was observed in both males [0.237% (95% CI, 0.216 ~ 0.259%)] and females [0.197% (95% CI, 0.141 ~ 0.254%)], AAPC in the ASMR was observed in both males [-0.398% (95% CI, -0.605~-0.191%)] and females [-0.295% (95% CI, -0.424~-0.65%)]. Age effects indicated that the relative risk (RR) of RA-associated incidence and mortality rates increased with age among males and females. The RR of RA increased over time and started to gradually increase from 1990. Cohort effects showed decreases in incidence, mortality and DALY rates in successive birth cohorts. The global incidence of RA would continue to increase in the future, while mortality would continue to decrease.

**Conclusion:**

The increased risk of RA is dominantly influenced by age effects and period effects and the ethnic area. The results suggest that early identification and treatment of RA is important for reducing the ongoing burden with age, and targeted health education and specific intervention programs should be promoted to control middle-elderly population.

**Supplementary Information:**

The online version contains supplementary material available at 10.1186/s12890-023-02594-2.

## Background

Rheumatoid arthritis (RA) is a systemic autoimmune disease with symmetrical inflammatory polyarthritis as the main clinical manifestation [1]. It usually begins in the small joints of the hands and feet, then spreads to the larger joints, eventually leading to disabling joint destruction and premature mortality [2, 3]. Despite medicine advances, the disease burden of RA remains high. RA also causes many health care costs, as it has been an incurable disease until now. The world’s population is aging, with the World Health Organization (WHO) estimating that the number of individuals aged 65 years or older will increase from 524 million in 2010 to 1.5 billion in 2050 [4]. As a result of this increased longevity and a rising frequency of chronic disease risk factors, chronic disease prevalence is projected to rise steadily. The consequences are more severe and include death and disability, reduced quality of life, and increased health care utilization and costs [5]. This is particularly obvious in RA, a disease that may accelerate aging, portend poor long-term outcomes, and have significant financial implications [6–8]. It follows that RA is a major public health problem, associated with a substantial burden of functional disability.

Based on the analysis of previous disease burden data, forecasting the future incidence rate can better guide the prevention of RA and promote the rational treatment. To this end, this study described and analyzed global RA incidence, mortality and disability-adjusted of life years (DALYs) from 1990 to 2019. Meanwhile, an age-period-cohort model with an intrinsic estimator (IE) is established based on these data, and the incidence and mortality rates of RA in the next 30 years are predicted, which can provide a reference for the adjustment of global intervention and treatment policies for RA.

## Materials and methods

### Data sources

Contributed by the Institute for Health Metrics and Evaluation (IHME: http://www.healthdata.org/gbd/2019), the Global Burden of Disease 2019 (GBD 2019) aims to quantify the comparative magnitude of health loss due to diseases, injuries, and risk factors by age, sex, and geographies for specific points in a series of times. Estimates such as incidence, mortality and DALYs were conducted among groups stratified by age, sex and year on the GBD [9, 10]. The study provides an improved standardized methodology and comprehensive assessment of incidence, mortality and DALYs for 369 diseases and injuries and 87 risk factors in 204 countries and territories.

RA events were diagnosed and classified based on the International Statistical Classification of Diseases and Related Health Problems, Tenth Revision (ICD-10). For RA, ICD-10 codes are M05.3, M05.8, M05.9, M06.0, M06.8, M06.9 and M08.0. The modelling strategy for estimating RA incidence, mortality and DALYs was generalized by grouping countries based on quality and types of data available [11]. Our data on RA were obtained from the global health data exchange (GHDx) section result tools (https://vizhub.healthdata.org/gbd-results/). Ethical approval was not needed for this study because there was no direct involvement of human subjects.

### Data analysis

#### Join point regression analysis

The annual percentage change (APC) and the average annual percentage change (AAPC) for each segment were estimated by join point regression, which focused on estimating the temporal trends in the age-standardized incidence rate (ASIR), age-standardized mortality rate (ASMR) and age-standardized DALY rate of RA. We applied the join point regression program version 4.8.0.1 from the Statistical Research and Applications Branch of the Surveillance Research Program of the U.S. National Cancer Institute.

### APC analysis

This study used the APC framework to assess the incidence, mortality and DALY rates of RA globally and to assess the potential impact of age, period, and cohort effects on these trends. The APC model was developed based on the Poisson distribution, and can be generally expressed as follows [12].

Y = log*(M) =µ* + *α* age_*j*_ + *β* period_*j*_ + *γ* cohort_*j*_ + *ε*_i_, where M is the incidence, mortality, or DALY rate of the corresponding age group; *α*, *β*, and *γ* denote the coefficients of age, period, and cohort of the model, respectively; *µ* denotes the intercept of the model; and *ε*_i_ denotes the residual of the model.

The time series from 1990 to 2019 was chopped into six consecutive intervals. Successive 5-year age groups from 0 ~ 14 to 89 ~ 94 years were divided. Individuals younger than 9 and older than 95 were excluded. The risk ratio (RR) was the exponential value of the coefficient, which denoted the risk in a particular age, period, or birth cohort relative to the reference groups. The specific value of the middle group was selected to represent the whole group. All analyses were conducted by Stata 15 software (Stata Corp, College Station, TX, USA). The Wald chi-square test was utilized to calculate the significance of the estimable parameters and functions. All statistical tests were 2-sided, and the significance level considered was 5%. Model fitting was evaluated by deviance, SE coefficients, Akaike’s information criterion (AIC), and the Bayesian information criterion (BIC).

### Incidence and mortality rate predicted based on bayesian age-period cohorts

This study predicted the rate of incidence and mortality due to RA from 2019 to 2044 by running an APC analysis by sex using the Nordpred package in R, taking into account the changing rates and changing population structure, which has been fully demonstrated and recognized in previous studies.

## Results

### Global level

#### The incidence rate

The ASIR of RA showed a relatively stable trend from 12.208/100,000 (95% UI, 11.126 ~ 13.376) in 1990 to 13.001/100,000 (95% UI, 11.833 ~ 14.274) in 2019. Both sexes followed a similar pattern, but the rates were higher in females than in males across all groups. The ASIR increased from 7.590/100,000 (95% UI, 6.904 ~ 8.336) in 1990 to 8.133/100,000 (95% UI, 7.380 ~ 8.944) in 2019 for males, and from 16.79% (95% UI, 15.355 ~ 18.343%) in 1990 to 17.80/100,000 (95% UI, 16.218 ~ 19.523) in 2019 for females.

**Fig. 1:**
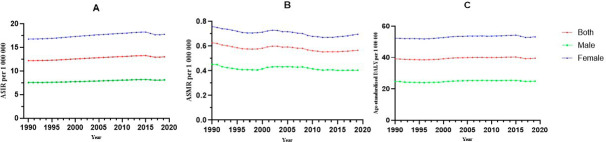
The trend of the RA in global between 1990 and 2019. (**A**): ASIR; (**B**): ASMR; (**C**): Age-standardized DALY rate

Before 2015, the ASIR of RA showed a slighter upward trend globally, and an opposite trend was observed after 2015. The ASIR decreased from 8.223/100,000 (95% UI, 7.488 ~ 9.014) in 2015 to 8.133/100,000 (95% UI, 7.380 ~ 8.944) in 2019 in males, and decreased from 18.272/100,000 (95% UI, 16.672 ~ 19.934) in 2015 to 17.715/100,000 (95% UI, 16.133 ~ 19.433) in 2018 in females (Fig. 1A).

### The mortality rate

The ASMR of RA showed a fluctuating downward trend, from 0.627/100,000 (95% UI, 0.491 ~ 0.775) in 1990 to 0.566/100,000 (95% UI, 0.431 ~ 0.660) in 2019. The ASMR decreased from 0.452/100,000 (95% UI, 0.354 ~ 0.499) in 1990 to 0.404/100,000 (95% UI, 0.350 ~ 0.457) in 2019 in males, and from 0.759/100,000 (95% UI, 1.029 ~ 0.553) in 1990 to 0.697/100,000 (95% UI, 0.465 ~ 0.859) in 2019 in females (Fig. 1B).

### The DALY rate

From 1990 to 2019, the age-standardized DALY rate showed a slightly fluctuating upward trend, from 39.116/100,000 (95% UI, 30.132 ~ 48.565) in 1990 to 39.565/100,000 (95% UI, 49.529 ~ 30.508) in 2019, which increased from 1.103% for males and 1.699% for females (Fig. 1C).

### Regional level

At the regional-level, the ASIR of RA was found to be highest in America, with 18.578 (95% UI, 17.147 ~ 20.148) in 2019. Whereas, Africa showed the lowest ASIR, with 9.351 (95% UI, 8.374 ~ 10.393) in 2019 (Table 1). The ASMR was found to be highest in Asia, with 0.681 (95% UI, 0.802 ~ 0.480) in 2019. However, Africa showed the lowest rate, with 0.348 (95% UI, 0.428 ~ 0.212) in 2019 (Table 1). The age-standardized DALY rate was found to be highest in America, with 53.676 (95% UI, 40.106 ~ 67.968). Africa showed the lowest age-standardized DALY rate, with 27.024 (95% UI, 20.471 ~ 34.619) (Table 1).

**Table 1:** Rate of RA in 1990 and 2019 and age-standardized rates by region (95% uncertainty interval)

	The incidence rate (per 100,000)		The mortality rate (per 100,000)		The DALY rate (per 100,000)	
	1990	2019	1990	2019	1990	2019
**Global**	12.208(11.126,13.376)	13.001(11.833, 14.274)	0.627(0.491,0.775)	0.566 (0.431,0.660)	39.116(30.132,48.565)	39.565(30.508, 49.529)
**Africa**	8.525(7.668,9.480)	9.351(8.374,10.393)	0.396(0.253,0.498)	0.348(0.428,0.212)	26.579(20.239, 34.083)	27.024(20.471, 34.619)
**America**	16.447(15.226,17.773)	18.578(17.147,20.148)	0.624(0.453,0.887)	0.567(0.810,0.414)	49.016(37.130, 61.573)	53.676(40.106,67.968)
**Asia**	12.142(11.032,13.364)	12.689(11.481,13.947)	0.701(0.574, 0.842)	0.681 (0.802,0.480)	38.865(30.208, 48.563)	39.580(30.733,49.292)
**Europe**	11.713(10.691,12.848)	12.770(11.659,14.039)	0.582(0.386,0.792)	0.360 (0.255,0.491)	39.594(30.039, 49.796)	37.434(27.660, 47.944)

**Fig. 2:**
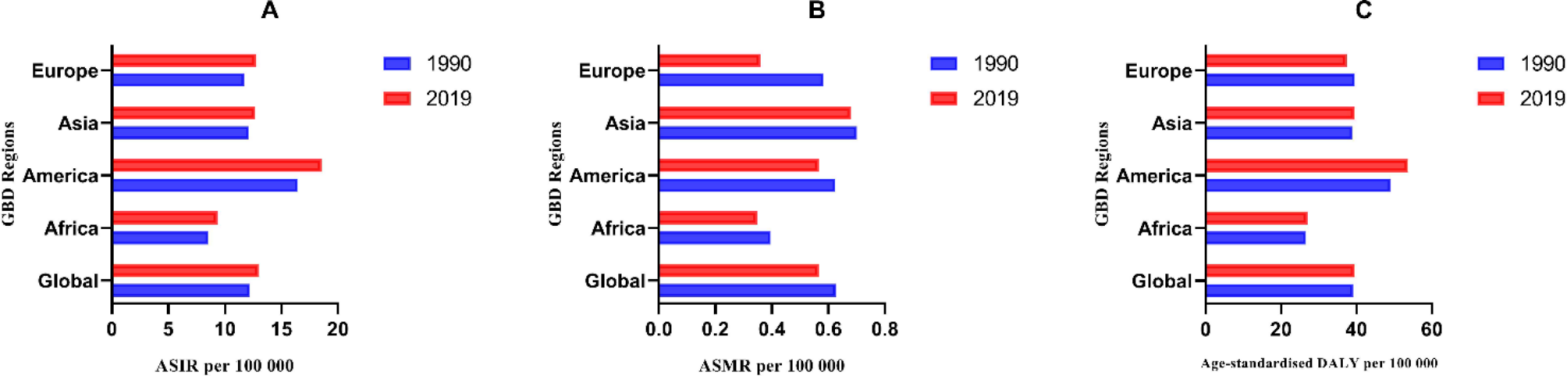
The trend of the RA in regional between 1990 and 2019. (**A**): ASIR; (**B**): ASMR; (**C**): Age-standardized DALY rate

The ASIR showed an increasing trend (Fig. 2A), while the ASMR showed a decreasing trend (Fig. 2B). Except for Europe, the age-standardized DALY rate showed an upward trend (Fig. 2C). The percentage change in regional-level ASIR, ASMR and age-standardized DALY rate estimates for all GBD 2019 regions are presented between 1990 and 2019 in online supplemental Figure S1.

### Join point regression analysis

Generally, the ASIR shown an overall fluctuating upward trend, but decreased for both males and females from 2014 to 2017. The AAPC in the whole period was 0.237% (95% CI, 0.216 ~ 0.259%) and 0.197% (95% CI, 0.141 ~ 0.254%) for males and females, respectively. The ASMR decreased from 2002 to 2019, except for a flat trend among males, during the periods of 2002 to 2008 and 2012 to 2019. While in females, the ASMR substantially decreased from 2002 to 2010 and then maintained a flat trend from 2010 to 2014. Throughout the whole period, the AAPC was − 0.398% (95% CI, -0.605~-0.191%) and 0.295% (95% CI, -0.424~-0.165%) for males and females, respectively.

**Fig. 3:**
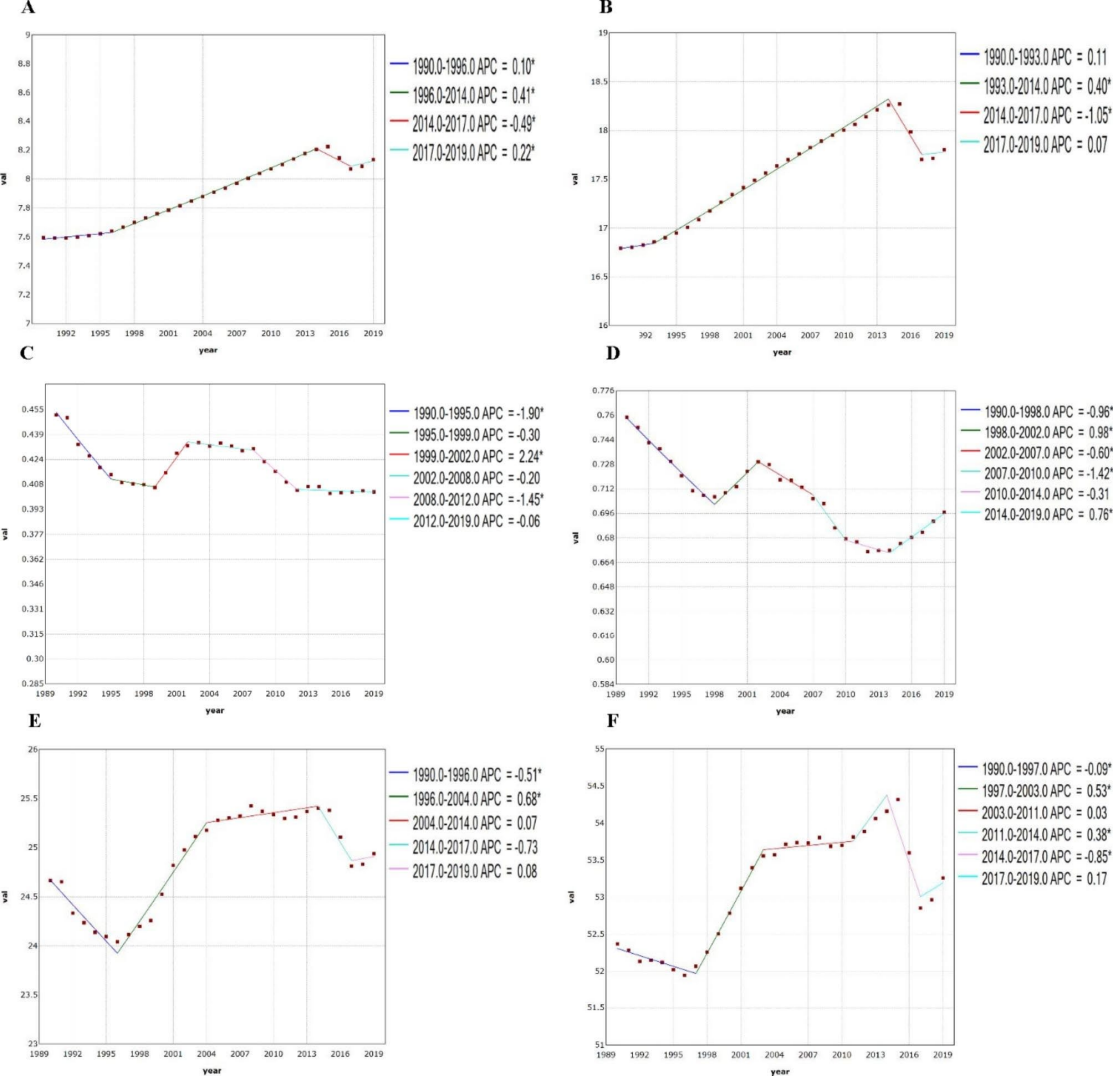
Join point regression analysis for RA between 1990 and 2019. (**A**): ASIR for male; (**B**): ASIR for female; (**C**): ASMR for male; (**D**): ASMR for female; (**E**): Age-standardized DALY rate for male; (**F**): Age-standardized DALY rate for female. *: Indicates that the APC is significantly different from zero at the alpha = 0.05 level

The age-standardized DALY rate substantially increased from 1996 to 2004 and then maintained a flat trend during the period from 2004 to 2014 in males. However, in females, the age-standardized DALY rate substantially increased from 1997 to 2003 and then maintained a flat trend during the period from 2003 to 2011. Throughout this period, the AAPC was 0.034% (95% CI, -0.072~-0.039%) and 0.058% (95% CI, 0.011 ~ 0.105%) for males and females, respectively. Join point regression analysis of the sex-specific, ASIR, ASMR and age-standardized DALY rate for RA in global from 1990 to 2019 is shown in Table 2; Fig. 3.

**Table 2:** AAPC and APC of Incidence, Mortality and DALY rate of RA among males and females

Categories	Males		Females	
	period	APC, % (95% UI)	period	APC, % (95% UI)
**Incidence rate**				
	1990–1996	0.100(0.070,0.130) **	1990–1993	0.106(-0.116,0.329)
	1996–2014	0.407(0.401,0.414) **	1993–2014	0.402(0.390,0.414) **
	2014–2017	-0.493(-0.670, -0.316) **	2014–2017	-1.046(-1.485, -0.605) **
	2017–2019	0.221(0.043,0.399) *	2017–2019	0.066(-0.378,0.512)
**AAPC, % (95% CI)**		0.237(0.216,0.259) **		0.197(0.141,0.254) **
**Mortality rate**	1990–1995	-1.899(-2.217, -1.579) **	1990–1998	-0.960(-1.057, -0.863) **
	1995–1999	-0.299(-1.021,0.429)	1998–2002	0.983(0.532,1.436) **
	1999–2002	2.240(0.764,3.737) *	2002–2007	-0.602(-0.883, -0.320) **
	2002–2008	-0.202(-0.525,0.123)	2007–2010	-1.416(-2.295, -0.529) *
	2008–2012	-1.449(-2.162, -0.730) *	2010–2014	-0.306(-0.752, 0.141)
	2012–2019	-0.055(-0.249,0.139)	2014–2019	0.757(0.556, 0.959) **
**AAPC, % (95% CI)**		-0.398(-0.605, -0.191) **		-0.295(-0.424, -0.165) **
**DALY rate**	1990–1996	-0.509(-0.648, -0.370) **	1990–1997	-0.092(-0.132, -0.053) **
	1996–2004	0.679(0.568, 0.790) **	1997–2003	0.528(0.462, 0.594) **
	2004–2014	0.066(-0.009, 0.142)	2003–2011	0.027(-0.012, 0.066)
	2014–2017	-0.733(-1.55, 0.090)	2011–2014	0.384(0.089, 0.679) *
	2017–2019	0.084(-0.740, 0.914)	2014–2017	-0.847(-1.137, -0.555) **
		0.034(-0.072, 0.139)	2017–2019	0.175(-0.119, 0.470)
**AAPC, %(95%CI)**		0.034(-0.072, 0.139)		0.058(0.011, 0.105) *

* p < 0.05, **p < 0.001

### APC analysis

#### Age effect

**Fig. 4:**
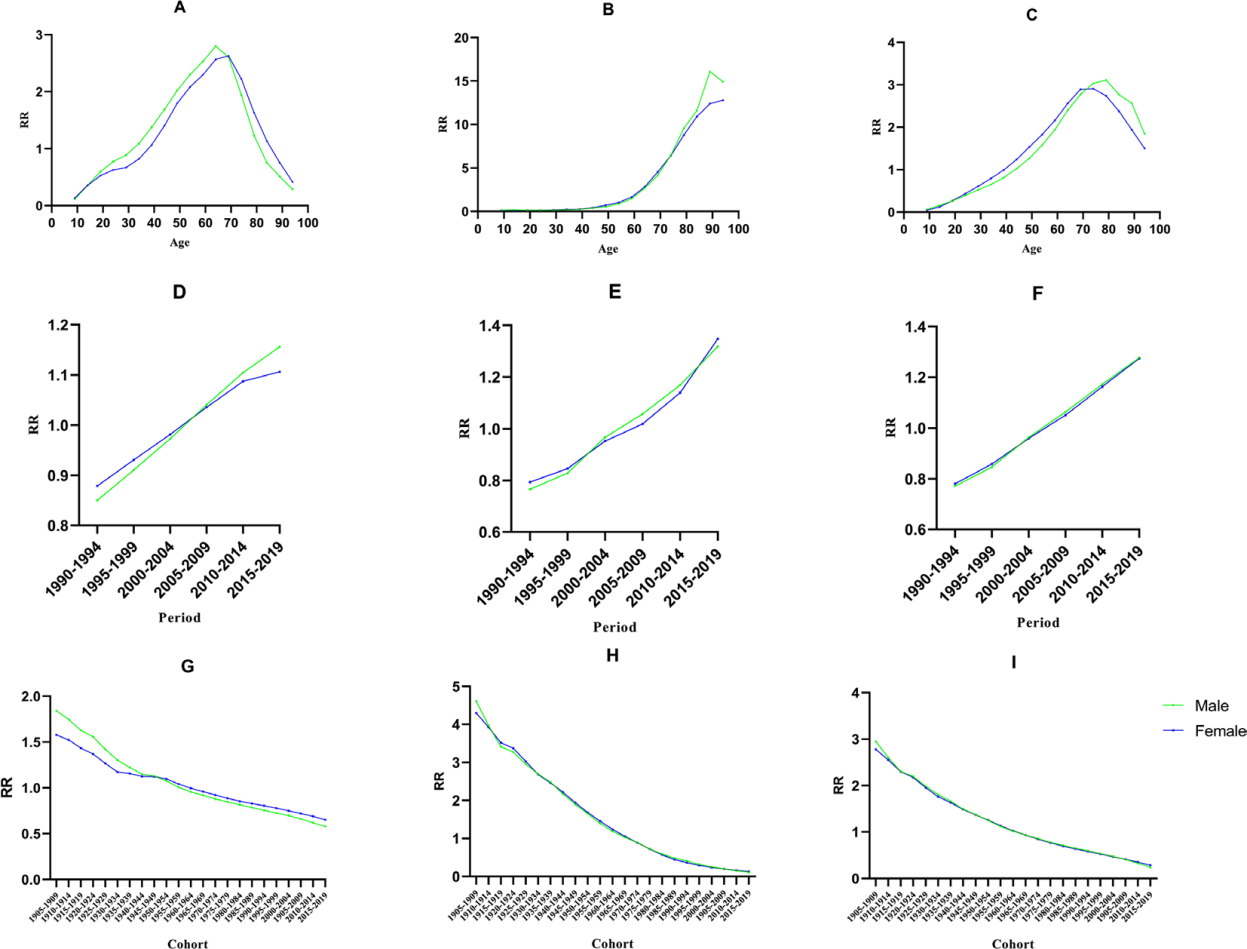
APC Analysis on RA for both sexes: Age effect on RA for both sexes. (**A**): ASIR; (**B**): ASMR; (**C**): Age-standardized DALY rate;Period effect on RA for both sexes. (**D**): ASIR; (**E**): ASMR; (**F**): Age-standardized DALY rate; Birth cohort effect on RA for both sexes. (**G**): ASIR; (**H**): ASMR; (**I**): Age-standardized DALY rate

The age effect on the ASIR of RA is shown in Table 3. The minimum age of the male RR was 0.137 (95% CI, 0.054 ~ 0.345) in the 4 ~ 9-year-old group, and the maximum age of the male RR was 2.630 (95% CI, 2.164 ~ 3.197) in the 65 ~ 69- year-old group. The highest RR was 2.799 (95% CI, 2.418 ~ 3.239) in the 60 ~ 64 age group, which was 24.129-fold higher than that in the 4 ~ 9 age group (Fig. 4A).

**Table 3:** Specific relative risk of RA incidence, mortality, and DALY rate due to age, period, and cohort effects

	ASIR		ASMR		Age-standardized DALY rate	
**Factors**	Male	Female	Male	Female	Male	Female
	RR(95%CI)	RR(95%CI)	RR(95%CI)	RR(95%CI)	RR(95%CI)	RR(95%CI)
**Age**						
4–9	0.137 (0.054, 0.345) **	0.116 (0.059, 0.229) **	0.143 (0, 4818.51)	0.112 (0, 1007.802)	0.065 (0.025, 0.167) **	0.045 (0.021, 0.099) **
10–14	0.360 (0.209, 0.621) **	0.356 (0.244, 0.519) **	0.167 (0, 652.024)	0.105 (0, 301.362)	0.157 (0.090, 0.273) **	0.125 (0.081, 0.192) **
15–19	0.527 (0.334, 0.832) *	0.597 (0.442, 0.807) *	0.124 (0, 633.349)	0.12 (0, 112.347)	0.265 (0.173, 0.406) **	0.268 (0.198, 0.364) **
20–24	0.627 (0.412, 0.953) *	0.773 (0.592, 1.011)	0.120 (0, 333.690)	0.125 (0, 59.512)	0.399 (0.281, 0.567) **	0.439 (0.343, 0.562) **
25–29	0.669 (0.447, 1.001)	0.890 (0.692, 1.143)	0.133 (0, 148.301)	0.155 (0.001, 28.374)	0.526 (0.386, 0.715) **	0.613 (0.495, 0.759) **
30–34	0.819 (0.566, 1.184)	1.091 (0.867, 1.373)	0.176 (0, 62.476)	0.218 (0.003, 15.768)	0.656 (0.498, 0.864) *	0.795 (0.657, 0.960) *
35–39	1.065 (0.764, 1.486)	1.377 (1.117, 1.699) *	0.239 (0.002, 35.382)	0.264 (0.006, 11.313)	0.816 (0.637, 1.045)	0.993 (0.838, 1.176)
40–44	1.404 (1.042, 1.893) *	1.691 (1.395, 2.050) **	0.383 (0.007, 22.212)	0.418 (0.020, 8.735)	1.023 (0.822, 1.274)	1.240 (1.068, 1.440) *
45–49	1.800 (1.377, 2.353) **	2.023 (1.695, 2.416) **	0.536 (0.018, 15.93)	0.716 (0.063, 8.119)	1.268 (1.046, 1.536) *	1.540 (1.352, 1.755) **
50–54	2.083 (1.630, 2.662) **	2.301 (1.951, 2.713) **	0.864 (0.057, 12.981)	1.017 (0.140, 7.393)	1.581 (1.339, 1.867) **	1.827 (1.630, 2.046) **
55–59	2.297 (1.832, 2.879) **	2.534 (2.170, 2.959) **	1.467 (0.177, 12.163)	1.651 (0.348, 7.824)	1.941 (1.683, 2.237) **	2.163 (1.961, 2.385) **
60–64	2.569 (2.089, 3.160) **	2.799 (2.418, 3.239) **	2.692 (0.534, 13.568)	2.848 (0.853, 9.514)	2.405 (2.131, 2.715) **	2.564 (2.356, 2.790) **
65–69	2.630 (2.164, 3.197) **	2.613 (2.260, 3.022) **	4.157 (1.118, 15.465) *	4.548 (1.67, 12.385) *	2.775 (2.494, 3.087) **	2.895 (2.685, 3.122) **
70–74	2.226 (1.825, 2.715) **	1.945 (1.665, 2.273) **	6.464 (1.865, 22.399) *	6.432 (2.371, 17.449) **	3.035 (2.744, 3.357) **	2.910 (2.704, 3.132) **
75–79	1.630 (1.312, 2.025) **	1.231 (1.029, 1.474) *	9.551 (2.284, 39.936) *	8.787 (2.705, 28.539) **	3.111 (2.801, 3.456) **	2.741 (2.535, 2.963) **
80–84	1.134 (0.885, 1.452)	0.752 (0.606, 0.932) *	11.608 (1.932, 69.751) *	10.909 (2.498, 47.65) *	2.772 (2.460, 3.123) **	2.380 (2.178, 2.600) **
85–89	0.754 (0.564, 1.008)	0.512 (0.399, 0.658) **	16.086 (1.716, 150.776) *	12.400 (1.991, 77.238) *	2.572 (2.240, 2.952) **	1.943 (1.752, 2.154) **
90–94	0.415 (0.282, 0.611) **	0.291 (0.208, 0.406) **	14.926 (0.971, 229.339)	12.807 (1.397, 117.42) *	1.846 (1.563, 2.180) **	1.504 (1.329, 1.701) **
**Period**						
1990–1994	0.850 (0.737, 0.98) *	0.879 (0.797, 0.970) *	0.766 (0.183, 3.208)	0.793 (0.261, 2.416)	0.771 (0.704, 0.844) **	0.781 (0.732, 0.832) **
1995–1999	0.910 (0.799, 1.038)	0.931 (0.849, 1.021)	0.828 (0.339, 2.021)	0.846 (0.422, 1.694)	0.846 (0.789, 0.907) **	0.857 (0.816, 0.901) **
2000–2004	0.973 (0.858, 1.103)	0.982 (0.898, 1.073)	0.967 (0.636, 1.472)	0.952 (0.684, 1.325)	0.963 (0.909, 1.021)	0.959 (0.920, 1.000)
2005–2009	1.040 (0.917, 1.180)	1.035 (0.947, 1.132)	1.057 (0.692, 1.614)	1.018 (0.728, 1.425)	1.063 (1.002, 1.128)	1.050 (1.006, 1.096) *
2010–2014	1.105 (0.971, 1.257)	1.087 (0.993, 1.19) *	1.169 (0.48, 2.847)	1.140 (0.569, 2.284)	1.173 (1.094, 1.257) **	1.163 (1.107, 1.223) **
2015–2019	1.156 (1.011, 1.323) *	1.106 (1.007, 1.216) *	1.319 (0.32, 5.432)	1.348 (0.448, 4.054)	1.277 (1.174, 1.390) **	1.275 (1.200, 1.354) **
**Birth Cohort**						
1900–1904	1.841 (0.796, 4.26)	1.578 (0.752, 3.315)	4.612 (0.147, 144.899)	4.297 (0.285, 64.841)	2.947 (2.236, 3.885) **	2.779 (2.255, 3.425) **
1905–1909	1.747 (1.03, 2.964) *	1.524 (0.958, 2.422)	3.969 (0.215, 73.443)	3.928 (0.393, 39.214)	2.600 (2.092, 3.232) **	2.551 (2.165, 3.006) **
1910–1914	1.629 (1.091, 2.431) *	1.434 (1.010, 2.037) *	3.409 (0.297, 39.179)	3.517 (0.511, 24.186)	2.303 (1.911, 2.776) **	2.302 (2.001, 2.649) **
1915–1919	1.558 (1.128, 2.152) *	1.370 (1.037, 1.81) *	3.272 (0.436, 24.539)	3.373 (0.684, 16.629)	2.200 (1.868, 2.591) **	2.178 (1.924, 2.465) **
1920–1924	1.42 (1.077, 1.874) *	1.268 (1.004, 1.602) *	2.958 (0.555, 15.773)	3.025 (0.802, 11.415)	1.986 (1.711, 2.306) **	1.956 (1.747, 2.191) **
1925–1929	1.303 (1.016, 1.67) *	1.174 (0.956, 1.442)	2.698 (0.621, 11.721)	2.686 (0.839, 8.597)	1.806 (1.568, 2.079) **	1.760 (1.581, 1.959) **
1930–1934	1.224 (0.963, 1.557)	1.157 (0.954, 1.403)	2.487 (0.566, 10.931)	2.465 (0.769, 7.897)	1.665 (1.444, 1.921) **	1.634 (1.466, 1.821) **
1935–1939	1.151 (0.903, 1.466)	1.126 (0.933, 1.359)	2.171 (0.396, 11.900)	2.220 (0.592, 8.333)	1.496 (1.285, 1.743) **	1.490 (1.330, 1.669) **
1940–1944	1.129 (0.882, 1.445)	1.124 (0.934, 1.353)	1.899 (0.241, 14.941)	1.939 (0.393, 9.563)	1.375 (1.164, 1.623) **	1.370 (1.212, 1.549) **
1945–1949	1.077 (0.832, 1.394)	1.098 (0.911, 1.324)	1.655 (0.133, 20.541)	1.686 (0.242, 11.733)	1.250 (1.039, 1.504) *	1.257 (1.099, 1.438) *
1950–1954	1.006 (0.762, 1.326)	1.042 (0.859, 1.265)	1.401 (0.067, 29.278)	1.461 (0.142, 15.036)	1.122 (0.913, 1.380)	1.134 (0.977, 1.315)
1955–1959	0.956 (0.709, 1.291)	0.996 (0.812, 1.222)	1.192 (0.032, 44.361)	1.241 (0.078, 19.804)	1.019 (0.809, 1.283)	1.026 (0.870, 1.209)
1960–1964	0.919 (0.664, 1.271)	0.959 (0.772, 1.191)	1.031 (0.015, 73.179)	1.054 (0.041, 27.102)	0.936 (0.725, 1.209)	0.935 (0.780, 1.121)
1965–1969	0.879 (0.62, 1.248)	0.923 (0.733, 1.162)	0.892 (0.006, 126.256)	0.890 (0.021, 38.181)	0.858 (0.648, 1.137)	0.850 (0.696, 1.037)
1970–1974	0.848 (0.583, 1.233)	0.886 (0.694, 1.131)	0.729 (0.002, 225.012)	0.725 (0.010, 54.871)	0.779 (0.573, 1.060)	0.769 (0.619, 0.956)
1975–1979	0.817 (0.547, 1.219)	0.854 (0.659, 1.106)	0.593 (0.001, 407.845)	0.575 (0.004, 86.886)	0.710 (0.508, 0.993) *	0.698 (0.551, 0.885)
1980–1984	0.786 (0.513, 1.205)	0.829 (0.629, 1.092)	0.484 (0, 687.804)	0.446 (0.001, 145.058)	0.651 (0.452, 0.937) *	0.638 (0.492, 0.826)
1985–1989	0.755 (0.475, 1.201)	0.806 (0.597, 1.088)	0.411 (0, 1005.372)	0.367 (0.001, 210)	0.598 (0.400, 0.893) *	0.583 (0.438, 0.777) **
1990–1994	0.727 (0.430, 1.227)	0.779 (0.553, 1.096)	0.321 (0, 3807.187)	0.296 (0, 704.455)	0.538 (0.338, 0.857) *	0.528 (0.378, 0.736) **
1995–1999	0.699 (0.382, 1.28)	0.752 (0.503, 1.124)	0.254 (0, 17479.761)	0.240 (0, 3190.05)	0.478 (0.270, 0.845) *	0.471 (0.312, 0.711) **
2000–2004	0.663 (0.312, 1.411)	0.721 (0.432, 1.206)	0.202 (0, 139728.984)	0.201 (0, 22633.582)	0.411 (0.190, 0.889) *	0.415 (0.235, 0.733) *
2005–2009	0.621 (0.21, 1.835)	0.690 (0.322, 1.481)	0.152 (0, 5253578.387)	0.161 (0, 853550.511)	0.332 (0.099, 1.112)	0.356 (0.139, 0.908) *
2010–2014	0.579 (0.057, 5.871)	0.653 (0.117, 3.639)	0.110 (0, 6398086005975.39)	0.129 (0, 10756434816.291)	0.242 (0.015, 3.941)	0.288 (0.033, 2.504)
**Deviance**	0.140	0.124	0.065	0.097	0.798	1.399
**AIC**	4.804	5.475	2.288	2.591	5.929	6.632
**BIC**	-299.517	-299.532	-299.592	-299.559	-298.85889	-298.257

*RR, relative risk; CI, confidence interval; AIC, Akaike Information Criterion; BIC, Bayesian Information Criterion*

* p < 0.05, ** p < 0.001

The effect of age on RA mortality showed no significant difference in ASMR before 64 years of age. The age effect exhibited an increasing trend from 5 ~ 89 age in both sexes and reached the highest in the 85 ~ 89 age group of males, in which the RR was 16.086 (95% CI, 1.716 ~ 150.776), and reached the highest in the 90 ~ 94 age group of females, in which the RR was 12.807 (95% CI, 1.397 ~ 117.42) (Fig. 4B).

The trend on age-standardized DALY rate of the age effect was similar in both sexes. From the 9 ~ 14 age group to the 64 ~ 69 age group, which showed an upward trend, while from the 74 ~ 79 to 89 ~ 94 age groups, it dropped slightly (Figure 4C).

#### Period effect

The period effect on incidence, mortality and DALYs is shown in Fig. (4D-4F). RR for period effects was estimated separately after controlling for the other two effects. Both sexes had similar patterns of period effects in incidence, mortality and DALYs. Compared with 1994, the incidence rate in 2019 increased 1.361-fold for males and 1.259-fold for females. The effect of period on RA mortality rate showed no significant difference on ASMR. The age-standardized DALY rate of RR in 2019 was 1.277 (95% CI, 1.174 ~ 1.390) for males and 1.275 (95% CI, 1.200 ~ 1.354) for females.

#### Birth cohort effect

After controlling for the other two effects, the birth cohort RR revealed a slowly downward trend from the 1990 ~ 1995 cohort to the 2010 ~ 2014 cohort (Fig. 4G and I). In total, the incidence rate of the RA showed a slow downward trend, both in males and females, but with a lower level in males than in females after birth cohort 1945. However, the effect of cohort on the incidence rate of RA has not been significantly different since 1930. The cohort effect on mortality rate in RA is not significantly different across the entire interval. The influence of male and female cohorts on DALY rate of RA was similar in the whole interval.

### Morbidity and mortality projections

**Fig. 5:**
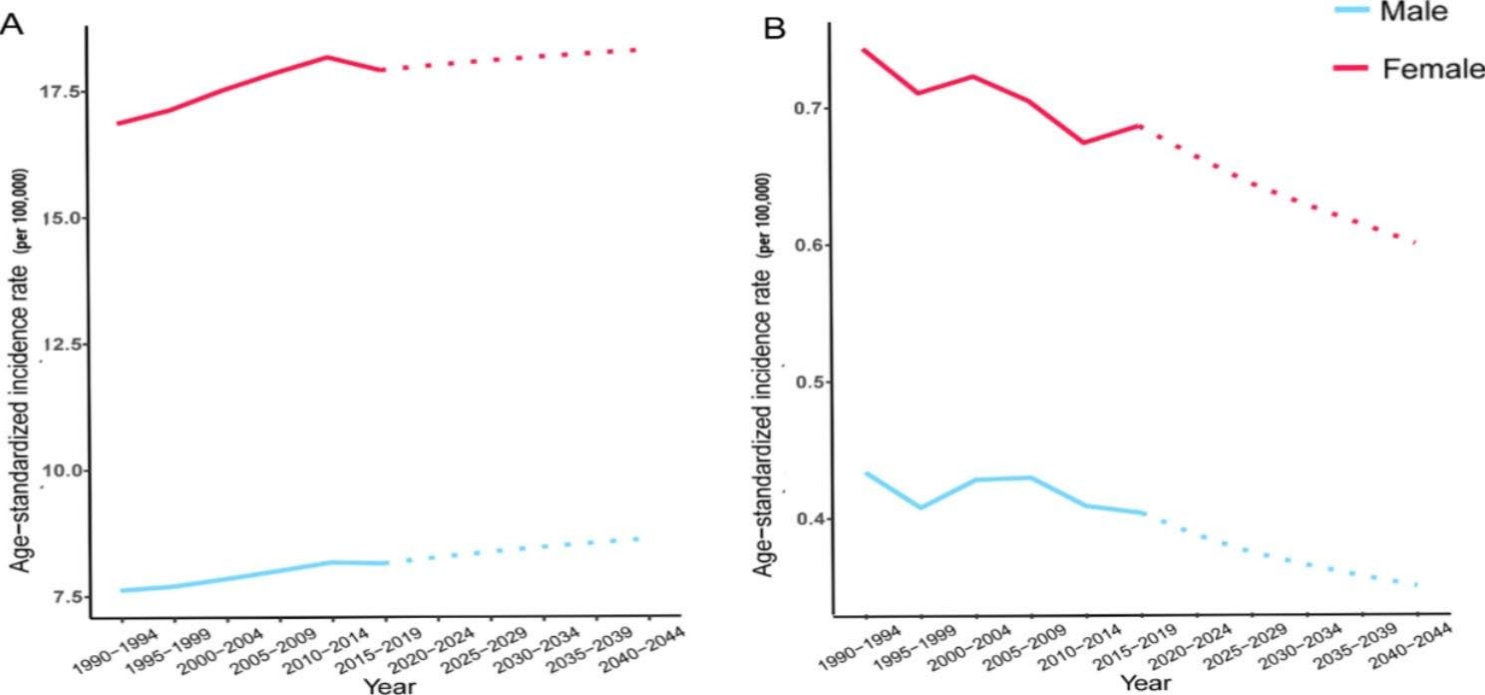
Forecasting global trends in RA. (**A**): ASIR; (**B**): ASMR

We predicted that the future incidence rate of RA would continue to increase while the mortality rate would continue to decrease globally, especially among males. Although the trends of incidence and mortality rates in females were somewhat similar to those in males, the incidence and mortality rates were substantially higher than those in males. Furthermore, the incidence rate was predicted to relatively stabilize after 2030, and the mortality rate was predicted to remain at a relatively low level, largely due to progress in a series of therapeutic progress and population increased awareness of the disease (Fig. 5).

## Discussion

The study explores the long-term trends in global RA ASIR, ASMR and age-standardized DALY rates of RA from 1990 to 2019 using the age-period-cohort framework based on data from the GBD 2019 study. The ASIR of RA showed a relatively stable trend from 12.208/100,000 (95% UI, 11.126 ~ 13.376) in 1990 to 13.001/100,000 (95% UI, 11.833 ~ 14.274) in 2019. The ASMR of RA showed a fluctuating downward trend. The age-standardized DALY rate showed a slight fluctuating upward trend. These data were consistent with the findings reported by W. Yang et al. [13]. Furthermore, we employed a join point regression analysis and developed an APC model to further investigate the incidence and DALY rate of RA. Both sexes followed a similar pattern, but the rates were higher in females than in males across all groups by join point regression analysis. APC with IE was established to analyze the influence of age, period and cohort effect on RA. The highest RR on the ASIR for males was 2.630 (95% CI, 2.164 ~ 3.197) in the 65 ~ 69 year-old-group. The future incidence of RA will increase, while the mortality rate will continue to decrease globally according to the model of prediction. These findings are of great help to epidemiologists and clinical experts in controlling the incidence trend of RA.

The minor decrease in the mortality is similar to that reported in a previous study in Global that investigated the period from 1990 to 2010 [14]. This may be the result of clear improvement in treatment strategies and more profound knowledge of the disease course. However, a previous global study examined trends in RA incidence and DALYs from 1990 to 2017 with inconsistent results [15]. The ASIR of RA increased by 8.2% between 1990 and 2017. Additionally, the age-standardized DALY rate of RA decreased by 3.6% between 1990 and 2017. This may be because we used the 2019 GBD database and they used the 2017 database. The updated data of two years will affect the previous algorithm.

In this study, the ASIR was higher among females than among males in 2019. This was consistent with previous studies [16]. The ASIR in females was 2.22 times the ASIR in males in 2015, which indicates that more females are affected by RA than males worldwide. This is consistent with previous studies [17]. The ASMR in females was also higher than that in males, which is in line with a higher incidence in females than in males, indicating that the female predominance is not simply due to the longer lifespan of females.

At the regional level, differences in incidence, mortality and DALY rates in regions should be noted. America has the highest incidence and DALY rates of RA, yet Asia has the highest mortality rate, and Africa has the lowest incidence, mortality and DALY rates. This might be due to regional, ethnic and economic differences. Therefore, more individualized guidelines for diagnosis and management may be needed.

According to the join point regression analysis, an increase in the age-standardized rate of incidence and DALYs related to RA represents an ongoing challenge. This suggests the importance of increased investment in the prevention and treatment of rheumatoid arthritis. However, there was an obvious downward trend from 2014 to 2017. It may be related to major events that had an impact on population changes during this period, such as the Ukrainian crisis in 2014, the European refugee crisis in 2015, and the Rohingya crisis in 2017, which resulted in many people being displaced and population migrations. The Ebola outbreak in 2014 also led to thousands of infections and deaths. Additionally, the approval of new therapies can contribute to changes in the ASIR and age-standardized DALY rates. For example, the 2012 American College of Rheumatology (ACR) guidelines [18] added three biologic agents, certolizumab, tocilizumab, and golimumab, and emphasized the importance of early disease control for preventing irreversible joint damage and maintaining long-term function. The 2015 ACR guidelines [19] for the first time included tofacitinib, a new small molecule drug for the first time. Meanwhile, the decreased in age-standardised rate of mortality related to RA suggests effective control. This may be related to effective treatment and early diagnosis [20]. Moreover, we also found that males had a higher incidence of AAPCs than females in the current study. This may reveal a potential increasing burden of RA in males. While males had lower AAPCs in the ASMR due to RA than females. This may reveal two points. First, the disease may progress more rapidly and be more aggressive in females; Second, male patients may respond better to treatment.

The age effect analysis showed that RA incidence consistently increased with advancing age from 9 to 64 years in females and from 9 to 69 years in males.The peak age at RA onset in both sexes was 49 ~ 69 years age group and 54 ~ 74 years age group, respectively. However, a previous study showed that worldwide mean age of RA onset was 44 ± 14 years (95% CI, 44 ~ 45) [21], which is slightly lower than our age. This finding indicates that RA incidence significantly increased with advancing age, mainly in older people, which might be attributable to the aging population globally. The age effect analysis showed that ASMR of RA consistently increased with advancing age from 9 to 94 years in both sexes. As we all known, the most common causes of mortality among RA patients are cardiovascular diseases, respiratory diseases, and infections [22]. The risk of cardiovascular disease and infection in RA patients increases greatly with age. This may be one reason for the increase in mortality with age.

The period effect analysis showed that ASIR, ASMR and age-standardized DALY rates of RA consistently increased over time. It is usually due to a series of complex historical events and environmental factors. This may be due to environmental changes and a more rapid pace of life. Lifestyle factors have changed substantially globally, including the adoption of unhealthy eating habits [23]. Previous studies have evaluated for several factors associated with the risk of RA in prior studies, including obesity [24], smoking, periodontitis [25], vitamin D deficiency [26] breast feeding and oral contraceptive use [27, 28].

The cohort effect on the ASIR, ASMR and age-standardized DALY rates due to RA continuously decreased in later birth cohorts in both males and females. The possible reason was that the later birth cohorts received better education and had a greater awareness of health and disease prevention than earlier birth cohorts [29, 30], as well as, the continuous update and improvement of treatment strategies.

The estimated increases in the incidence rate of RA and the numbers of new cases and deaths are expected to continue to increase globally during the next 25 years due to population growth and aging. Although the incidence and mortality trends were somewhat similar between both sexes, they were much higher in females than in males. Mortality rate is expected to remain at a relatively low level, mainly due to advances in treatment and improved the understanding of the disease among people.

However, there are some limitations that should be noted. First, although the GBD 2019 adapted numerous adjustments and corrections to the source, collation, and evaluation of the RA incidence, mortality and DALY rates to enhance data accuracy and comparability, it was undeniable that certain deviations in the completeness and accuracy of the GBD data were inevitable. Second, our study lacked an analysis of the comparison of RA incidence, mortality and DALY rates in the different national and different disease subtypes. Third, similar to other studies based on a population level, the APC-IE framework concluded the results at the population level. Ecological fallacy might occur because the study might not focus on the individual level. Therefore, more relevant studies focusing on these limitations should be carried out in the future to reveal more accurate analysis results.

## Conclusions

In summary, the ASIR of RA increased in globally for both males and females during the 1990 ~ 2019 period, with the peak age in the group aged 49 ~ 74 years. Due to the increase in high-risk behaviors and demographic changes in the coming decades, the incidence and mortality of RA worldwide will continue to increase. According to the regional level trend, the age-standardized incidence and DALY rates are increasing, especially in America. Therefore, an enhanced understanding of risk profiles and morbidity patterns associated with RA could facilitate the early identification of individuals, thereby reducing the future burden of this disease and better monitoring of disease burden and health outcomes. Meanwhile, more individualized guidelines for diagnosis and management may be needed.

### Electronic supplementary material

Below is the link to the electronic supplementary material.

Supplementary Material 1

## Data Availability

The datasets analyzed during the current study are available in the (Global Health Data Exchange) query tool produced by the IHME repository, [http://ghdx.healthdata.org/gbd-results-tool].

## Abbreviations

AAPC: Average Annual Percent change
APC: Annual Percent change
ASIR: Age Standardized Incidence Rate
ASMR: Age Standardized Mortality Rate
DALY: Disability-adjusted life year
GBD: Global Burden of Disease (study)
GHDx: Global Health Data exchange
ICD: International Classification of Disease
RR: Relative Risk
RA: Rheumatoid arthritis
WHO: World Health Organization
AIC: Akaike’s information criterion
BIC: Bayesian information criterion
IE: Intrinsic estimator
UI: Uncertainty interval

## References

[1] Sherrer YS, Bloch DA, Mitchell DM, Young DY, Fries JF (1986). The development of disability in rheumatoid arthritis. Arthritis Rheum.

[2] England BR, Sayles H, Michaud K (2016). Cause-specific mortality in male US veterans with rheumatoid arthritis. Arthritis Care Res.

[3] Sparks JA, Chang SC, Liao KP (2016). Rheumatoid arthritis and mortality among women during 36 years of prospective follow-up: results from the nurses’ health study. Arthritis Care Res.

[4] Fitzmaurice C, Abate D, Abbasi N (2019). Et al;for the global burden of Disease Cancer Collaboration.Global, Regional, and National Cancer incidence, mortality, years of Life Lost, Years lived with disability, and disability-adjusted life-years for 29 Cancer groups, 1990 to 2017: a systematic analysis for the global burden of Disease Study. JAMA Oncol.

[5] Salive ME (2013). Multimorbidity in older adults. Epidemiol Rev.

[6] Crowson CS, Liang KP, Therneau TM, Kremers HM, Gabriel SE (2010). Could accelerated aging explain the excess mortality in patients with seropositive rheumatoid arthritis?. Arthritis Rheum.

[7] Goronzy JJ, Shao L, Weyand CM (2010). Immune aging and rheumatoid arthritis. Rheum Dis Clin North Am.

[8] Birnbaum H, Pike C, Kaufman R, Marynchenko M, Kidolezi Y, Cifaldi M (2010). Societal cost of rheumatoid arthritis patients in the US. Curr Med Res Opin.

[9] Gregory Roth LB et al. Marczak,Kalkidan Hassen Abate, Global, regional, and national age-sex-specific mortality for 282 causes of death in 195 countries and territories, 1980–2017: a systematic analysis for the Global Burden of Disease Study 2017. Lancet. (2018) 392:1736–88. 10.1016/S0140-6736(18)32203-7 PMC6227606 30496103

[10] Wu D, Wong P, Guo C, Tam LS, Gu J (2021). Pattern and trend of five major musculoskeletal disorders in China from 1990 to 2017: findings from the global burden of Disease Study 2017. BMC Med.

[11] Jin Z, Wang D, Zhang H (2020). Incidence trend of five common musculoskeletal disorders from 1990 to 2017 at the global, regional and national level: results from the global burden of disease study 2017. Ann Rheum Dis.

[12] Dhamnetiya D, Patel P, Jha RP, Shri N, Singh M, Bhattacharyya K (2021). Trends in incidence and mortality of tuberculosis in India over past three decades: a joinpoint and age-period-cohort analysis. BMC Pulm Med.

[13] Yang W, Xu Y, Lin J et al. Global, regional and national burden of rheumatoid arthritis, and attributable risk factors from 1990 to 2019: update from the global burden of Disease 2019 study. Clin Exp Rheumatol. 2022 Dec 5. 10.55563/clinexprheumatol/h9lvvc 36533984

[14] Cross M, Smith E, Hoy D (2014). The global burden of rheumatoid arthritis: estimates from the global burden of disease 2010 study. Ann Rheum Dis.

[15] Safiri S, Kolahi AA, Hoy D (2019). Global, regional and national burden of rheumatoid arthritis 1990–2017: a systematic analysis of the global burden of disease study 2017. Ann Rheum Dis.

[16] Finckh A, Gilbert B, Hodkinson B (2022). Global epidemiology of rheumatoid arthritis Nat Rev Rheumatol.

[17] Megha Arora E, Gakidou Y, He (2018). Global, regional, and national age-sex-specific mortality and life expectancy, 1950–2017: a systematic analysis for the global burden of Disease Study 2017. Lancet.

[18] Singh JA, Furst DE, Bharat A (2012). 2012 update of the 2008 American College of Rheumatology recommendations for the use of disease-modifying antirheumatic drugs and biologic agents in the treatment of rheumatoid arthritis. Arthritis Care Res (Hoboken).

[19] Singh JA, Saag KG, Bridges SL (2016). 2015 American College of Rheumatology Guideline for the treatment of rheumatoid arthritis. Arthritis Rheumatol.

[20] Aletaha D, Smolen JS (2018). Diagnosis and management of rheumatoid arthritis: a review. JAMA.

[21] GEO-RA Group (2017). Latitude gradient influences the age of onset of rheumatoid arthritis: a worldwide survey. Clin Rheumatol.

[22] Otón T, Carmona L (2019). The epidemiology of established rheumatoid arthritis. Best Pract Res Clin Rheumatol.

[23] Myasoedova E, Davis J, Matteson EL (2020). Is the epidemiology of rheumatoid arthritis changing? Results from a population-based incidence study, 1985–2014. Ann Rheum Dis.

[24] Myasoedova E, Davis J, Matteson EL, Crowson CS (2013). Contribution of obesity to the rise in incidence of rheumatoid arthritis. Arthritis Care Res.

[25] Chou YY, Lai KL, Chen DY, Lin CH, Chen HH (2015). Rheumatoid arthritis risk associated with periodontitis exposure: a nationwide, population-based cohort study. PLoS ONE.

[26] Merlino LA, Curtis J, Mikuls TR, Cerhan JR, Criswell LA, Saag KG (2004). Vitamin D intake is inversely associated with rheumatoid arthritis: results from the Iowa women’s health study. Arthritis Rheum.

[27] Karlson EW, Mandl LA, Hankinson SE, Grodstein F (2004). Do breast-feeding and other reproductive factors influence future risk of rheumatoid arthritis? Results from the nurses’ health study. Arthritis Rheum.

[28] Doran MF, Crowson CS, O’Fallon WM, Gabriel SE (2004). The effect of oral contraceptives and estrogen replacement therapy on the risk of rheumatoid arthritis: a population based study. J Rheumatol.

[29] Jack Fox SL, James KJ, Krohn K, Lau B, Miltz, Christopher JL, Murray (2018). Global, regional, and national incidence, prevalence, and years lived with disability for 354 diseases and injuries for 195 countries and territories, 1990–2017: a systematic analysis for the global burden of Disease Study 2017. Lancet.

[30] Megha Arora M, Biehl S, Deiparine C, Ikeda GR, Kemp (2018). Hmwe Kyu. Global, regional, and national disability-adjusted life-years (DALYs) for 359 diseases and injuries and healthy life expectancy (HALE) for 195 countries and territories, 1990–2017: a systematic analysis for the global burden of Disease Study 2017. Lancet.

