# Supplementary figures and images for: Secular trend of mortality and incidence of rheumatoid arthritis in global ,1990–2019: an age period cohort analysis and joinpoint analysis
Source: BMC Pulm Med. 2023 Sep 22;23:356. doi: 10.1186/s12890-023-02594-2 (PMC10515246; doi:10.1186/s12890-023-02594-2)

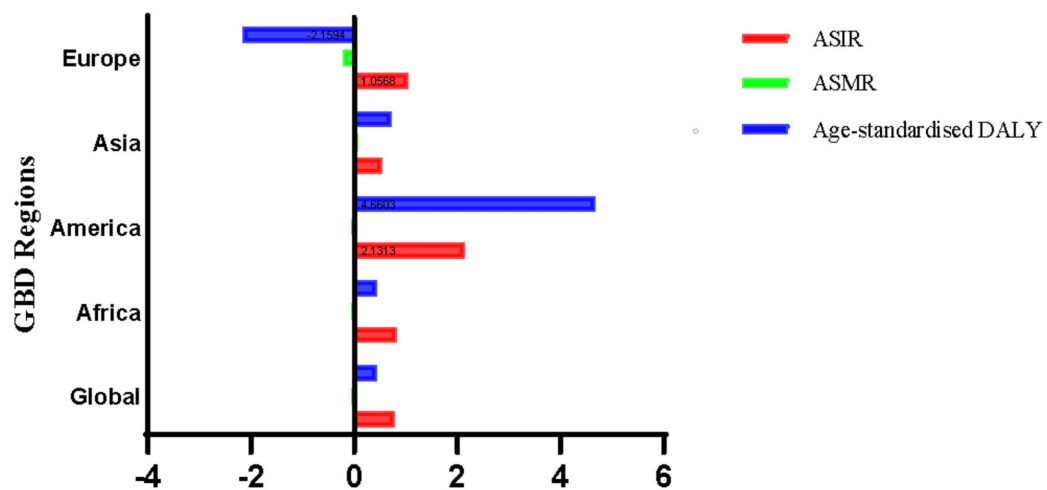

**Figure S1.** Percentage change between 1990 and 2019.

Supplement: Supplementary file 1 — Supplementary Material 1 [file 12890_2023_2594_MOESM1_ESM.pdf]
